# UPRmt-regulated mitokines: novel strategies for myocardial injury repair

**DOI:** 10.3389/fcell.2025.1652353

**Published:** 2025-10-29

**Authors:** Weinan Gao, Jia Liu, Wenda Zhang, Bin Liu, Luyan Shen

**Affiliations:** ^1^ Key Laboratory of Pathobiology, Department of Pathophysiology, Ministry of Education, College of Basic Medical Sciences, Jilin University, Changchun, China; ^2^ Department of Cardiology, The Second Hospital of Jilin University, Changchun, China

**Keywords:** mitochondrial stress, mitochondrial unfolded protein response (UPRmt), mitokines, fibroblast growth factor 21 (FGF21), cardiac diseases

## Abstract

Cardiac mitochondria generate ATP, *via* oxidative phosphorylation (OXPHOS) to sustain continuous and forceful myocardial contraction, thereby meeting systemic metabolic demands. Mitochondrial biogenesis and energy metabolism depend on proteostasis, which can be disrupted by stressors such as hypoxia, leading to impaired cardiac function. As a result, the study of mitochondrial energy metabolism and proteostasis under stress has become a key focus in cardiovascular research. The mitochondrial unfolded protein response (UPRmt) plays a “double-edged sword” role—either protective or detrimental—depending on the type, intensity, and duration of the stressor. This has sparked interest in strategies aimed at enhancing its adaptive signaling while inhibiting maladaptive pathways. Acting as mediators of intercellular communication, mitokines may transmit local mitochondrial stress signals to mitochondria in distant cells and tissues. This review analyzes and summarizes the role of UPRmt in regulating mitochondrial factors and explores the mechanisms through which fibroblast growth factor 21 (FGF21), secreted by the liver and skeletal muscle, influences protein homeostasis in cardiac myocytes. These insights aim to offer new avenues for the development of targeted UPRmt therapies and rehabilitation strategies for heart diseases.

## 1 Introduction

Cardiac diseases such as acute myocardial infarction (MI), chronic heart failure (HF), and ischemia–reperfusion injury pose serious threats to human health, with mitochondrial dysfunction being a central pathological mechanism. As a highly energy-dependent organ, the heart contains limited ATP reserves and thus relies on mitochondria to continuously generate ATP *via* oxidative phosphorylation (OXPHOS) to sustain contractile function. Mitochondria occupy 30%–40% of cardiomyocyte volume and are distributed among myofibrils, beneath the sarcolemma, and around the nucleus. They contribute to energy metabolism and signal transduction through calcium ions (Ca^2+^), reactive oxygen species (ROS), and other molecules (Nguyen et al., 2019; Hinton et al., 2024). Studies have shown that ischemic heart disease–induced heart failure with reduced ejection fraction (HFrEF) and metabolic abnormality–induced heart failure with preserved ejection fraction (HFpEF)—such as that seen in hypertension and obesity—are associated with mitochondrial respiratory chain dysfunction, abnormal ROS accumulation, mitochondrial DNA (mtDNA) heteroplasmy, and impaired biogenesis (Karamanlidis et al., 2011; Duan et al., 2019; Peoples et al., 2019; Elorza and Soffia, 2021). Mitochondrial damage contributes to the progression of myocardial infarction and heart failure by activating pathways such as apoptosis/necrosis and the NLRP3 inflammasome (Halestrap et al., 2004; Wang et al., 2023). Consequently, increasing attention has been paid to mitochondria as therapeutic targets in cardiac diseases.

Cardiomyocyte metabolic dysfunction is closely associated with mitochondrial damage. The mitochondrial protein homeostasis in injured cardiomyocytes not only determines the mitochondrial function but also dictates the fate of mitochondria. Mitochondrial damage, such as oxidative stress, unfolded proteins, and impairment of the electron transport system, can disrupt mitochondrial protein import, thereby triggering the Mitochondrial Stress Response (MSR) in mammalian cells. The MSR coordinates a series of adaptive responses, including the activation of the Mitochondrial Unfolded Protein Response (UPRmt), through multi-layered protein quality control mechanisms (adjustments in protein production, folding, and clearance) to restore mitochondrial function and maintain cellular homeostasis (Higuchi-Sanabria et al., 2018; Smyrnias et al., 2019; Hetz et al., 2020). Research on UPRmt has become an indispensable part of cardiovascular disease research (Chang et al., 2021; Liu et al., 2022). The UPRmt activated by hemodynamic overload, neurohumoral stress, *etc.*, can mitigate the exacerbation of mitochondrial dysfunction in cardiomyocytes and prevent myocardial contractile failure caused by cardiac dysfunction (Wang et al., 2019). With the increasing understanding of the role of stress in heart disease, people have begun to pay attention to the impact of psychological stress, environmental stress, and even exercise stress on heart disease, especially the pathways by which stress affects mitochondrial function and its role in maintaining the homeostasis of cells, tissues, or organs (Canada et al., 2021).

After mitochondrial perturbations in specific tissues of *Caenorhabditis elegans*. (for example, in neurons), MSR also occurred in distal tissues (such as in intestine), resulting in systemic effects such as lifespan extension (Durieux et al., 2011), indicating that local mitochondrial stress can communicate with other mitochondria in distant cells and tissues. Mitochondrial stress-induced mitokines are considered as intercellular and inter-organismal communication molecules that play a critical role in maintaining cellular homeostasis (Zhang et al., 2024). Growing evidence suggests that mitokines induced by appropriate physical activity may help reduce metabolic risk factors associated with heart failure (HF). Regular moderate exercise and tailored dietary strategies have been shown to support healthy aging and aid in cardiac rehabilitation. Mitokines are increasingly recognized as key mediators of exercise-induced physiological responses, as well as targets for dietary interventions and potential modulators of longevity. Moreover, tissue-specific mitochondrial dysfunction in organs such as the liver or skeletal muscle can initiate a mitochondrial stress response (MSR) in remote organs by stimulating the secretion of FGF21, thereby modulating systemic metabolic homeostasis (Nunnari and Suomalainen, 2012; Kang et al., 2021). These findings suggest that mitokines may serve as an entry point for further investigation into the roles of UPRmt-related molecules in cardiac diseases such as heart failure.

## 2 Mitochondrial unfolded protein response (UPRmt) and cardiac diseases

Mitochondrial–nuclear communication is essential for maintaining cellular function under stress. Mitochondria produce ATP *via* the tricarboxylic acid (TCA) cycle and OXPHOS and are involved in vital cellular processes such as energy metabolism, which require tight coordination between the nuclear and mitochondrial genomes. Under stress conditions, the accumulation of misfolded mitochondrial proteins, respiratory chain dysfunction, and excessive ROS production disrupt mitochondrial proteostasis. This triggers the mitochondrial stress response (MSR) in mammalian cells—a feedback network mediated by both anterograde signaling (from the nucleus to the mitochondria) and retrograde signaling (from the mitochondria to the nucleus). This bidirectional communication regulates protein homeostasis and mitochondrial quality control to repair or eliminate damaged organelles and maintain energy metabolic balance (Quirós et al., 2016; Naresh and Haynes, 2019).

The mitochondrial stress response (MSR) encompasses multiple response patterns that enhance mitochondrial adaptability and multifunctionality through the coordinated activation of several stress-response pathways (Quirós et al., 2016). As the first stress-protective response, UPRmt activates protein refolding or removes misfolded proteins to resist mitochondrial damage-mediated imbalance of protein homeostasis, which is considered the initial defense mechanism for cells to resist external stress (Sun et al., 2024). Researchers have identified four main UPRmt axes: the transcriptional canonical UPRmt axis, the mitochondrial intermembrane space (IMS) UPRmt axis, the translational canonical UPRmt axis, and the Sirtuin UPRmt axis (Cilleros-Holgado et al., 2023). Although the component molecules in different axes vary, these different UPRmt axes may be activated simultaneously and coordinate with each other, thus forming a complete UPRmt functional network. As an adaptive transcriptional response, the UPRmt is a retrograde signal from the mitochondria to the nucleus (Anderson and Haynes, 2020; Cilleros-Holgado et al., 2023), after which the nucleus sends new instructions to the mitochondria, forming a feedback regulatory loop.

The main mechanism of the classical UPRmt is as follows: when mitochondrial proteins are misfolded or protein import disorders, ATF5 cannot enter the mitochondria and initiate protective gene transcription. ATF5 is translocated to nucleus, where it activates the transcription of mitochondrial chaperones (such as mtHsp70, Hsp60, and Hsp10), mitochondrial proteases (such as ClpP, LonP1, OMI/HTRA2, paraplegin, YME1L, MPP, and OMA1), and antioxidants (thioredoxin 2), *etc.* (Dietl and Maack, 2017; Svaguša et al., 2020; Sun et al., 2024). Other UPRmt effectors, such as CHOP and ATF4, are also involved in the integrated stress response (ISR), another crucial component of the MSR. The ISR senses various stress signals through four specific kinases (PERK, GCN2, PKR, HRI), which regulate the phosphorylation of the translation initiation factor eIF2α. This phosphorylation suppresses global protein synthesis (Costa-Mattioli and Walter, 2020; Guo et al., 2020; Urbina-Varela et al., 2020; Ryoo, 2024), while selectively promoting the translation of transcription factors such as ATF4, CHOP, and ATF5. These transcription factors, in turn, regulate the expression of LONP1, ClpP, and YME1L, which cleave or process damaged or irreparable proteins not managed by HSPs (Palam et al., 2011; Svaguša et al., 2020).

**FIGURE 1:**
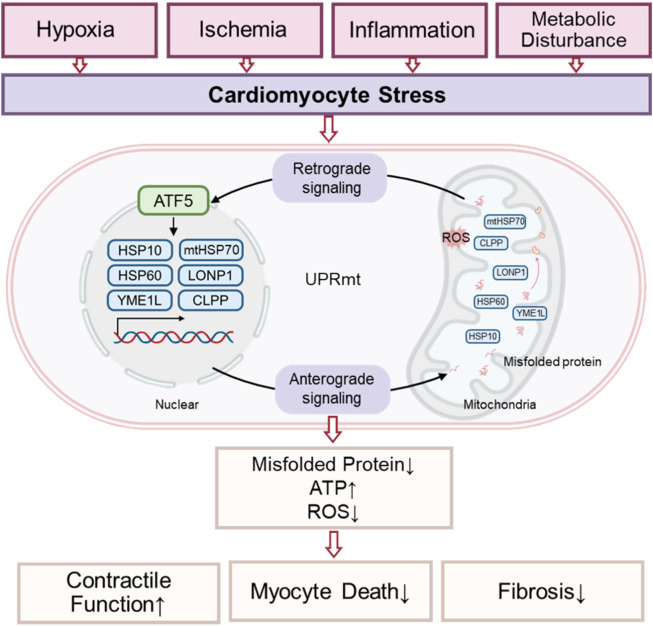
Hypothetical model of UPRmt signaling transduction in cardiomyocytes.

Although perspectives vary regarding the relationship between the ISR and UPRmt, the prevailing view is that ISR activation is essential for UPRmt function in mammals. Specifically, translation attenuation *via* ISR is a prerequisite for the transcriptional response to mitochondrial dysfunction (Pakos-Zebrucka et al., 2016; Quirós et al., 2017; Samluk et al., 2019). Evidence indicates that HRI, a cytoplasmic eIF2α kinase, mediates ISR activation following mitochondrial impairment. Mitochondrial dysfunction activates the metalloprotease OMA1, which cleaves DELE1. The cleaved DELE1 then translocates to the cytoplasm, oligomerizes, and binds directly to cytoplasmic HRI, stimulating eIF2α phosphorylation and thereby initiating the ISR (Fessler et al., 2020; Guo et al., 2020; Fessler et al., 2022). This pathway, as an indispensable component of UPRmt, maintains cellular homeostasis and restores mitochondrial function in response to stress. Moreover, mitochondrial dysfunction can reduce cytoplasmic aspartate and asparagine levels, which promotes GCN2-mediated eIF2α phosphorylation and triggers associated metabolic shifts (Mick et al., 2020; Misra et al., 2021). As a component of the adaptive transcriptional arm of UPRmt, the ISR attenuates global protein synthesis while allowing preferential translation of the transcription factors CHOP, ATF4, and ATF5 through upstream open reading frames (uORFs) (Vattem and Wek, 2004; Zhou et al., 2008; Quirós et al., 2017; Lu et al., 2022). This coordination of gene transcription and protein translation enables UPRmt and ISR to collaboratively regulate mitochondrial protein synthesis, folding, and degradation, ultimately restoring cellular homeostasis (Lu et al., 2022). These findings indicate that UPRmt acts as a central hub integrating multiple stress response pathways. Exploring UPRmt in cardiomyocytes is particularly valuable for understanding mitochondrial stress responses and their functional implications in cardiac pathophysiology (Figure 1).

Research into UPRmt during cardiac pathology is rapidly expanding. UPRmt activation, triggered by hemodynamic overload and neurohumoral stress, can mitigate mitochondrial dysfunction in cardiomyocytes and prevent contractile failure associated with heart disease. ATF5, a key transcription factor in UPRmt, has been shown to contribute to cardioprotection (Wang et al., 2019). Additionally, studies indicate that under hypoxic conditions, the UPRmt-induced protease LonP1 reduces ROS levels by degrading misfolded proteins and modulates mitochondrial bioenergetics, thereby exerting protective effects on the heart (Kuo et al., 2015; Venkatesh et al., 2019). However, prolonged or severe oxidative stress decreases LonP1 activity, which disrupts respiratory chain function and leads to left ventricular systolic dysfunction (Hoshino et al., 2014). Wai et al. reported that cardiac-specific deletion of YME1L, another downstream effector of UPRmt, results in the development of heart failure (Wai et al., 2015). Moreover, downregulation of the UPRmt-regulated endonuclease G causes excessive ROS production, impairs mtDNA replication, and induces cardiac hypertrophy in rodents (Blasco et al., 2018). UPRmt also facilitates the clearance and degradation of misfolded proteins in damaged mitochondria following myocardial infarction (MI), further supporting its role in cardiac function regulation.

Clinical data show that patients with high myocardial expression of UPRmt markers (ATF5, Hsp60, LonP1) exhibit significantly reduced myocardial fibrosis and lower cardiomyocyte mortality rates (Smyrnias et al., 2019). Mitochondria-targeted drugs, such as oligomycin, have been shown to alleviate lipopolysaccharide-induced cardiac dysfunction by specifically activating UPRmt (Wang Y. et al., 2021). Patients with low expression of HSP10, HSP60, HTRA2, OMA1, SPG7, and YME1L who have ischemic cardiomyopathy, dilated cardiomyopathy, or both require earlier heart transplantation or left ventricular assist device support (Bakovic et al., 2025). These findings suggest that appropriate activation of UPRmt may suppress myocardial injury. Although many studies have demonstrated that UPRmt activation promotes mitochondrial repair, enhances innate immune responses against pathogens, supports metabolic adaptation, and even extends lifespan, its role remains controversial. Some studies suggest that chronic or excessive UPRmt activation can trigger pro-inflammatory and apoptotic pathways, worsening tissue damage and accelerating heart disease progression (Kuo et al., 2015; Wang et al., 2016). Thus, UPRmt is considered a “double-edged sword,” and further research is needed to elucidate strategies for enhancing its beneficial effects to protect cardiac function.

## 3 Mitokines

Under stress conditions, specific organs release signaling molecules that act as mediators of intercellular communication, regulating systemic homeostasis *via* autocrine, paracrine, or especially endocrine pathways (Herrlich et al., 2022). Mitochondrial stress can trigger the release of distinct molecules into the circulation, which subsequently influence mitochondrial biology in distant target tissues to coordinate systemic responses. These molecules are referred to as *mitokines* (Durieux et al., 2011). Mitokines, secreted in response to mitochondrial stress or the mitochondrial unfolded protein response (UPRmt secreted in response to mitochondrial stress or UPRmt, facilitate interorgan crosstalk and coordinate metabolic regulation (Kim et al., 2013; Keipert et al., 2014). They primarily include signaling molecules encoded by nuclear DNA (e.g., GDF15 and FGF21) and those encoded by mitochondrial DNA (e.g., humanin, HN). Circulating mitokine levels are associated with aging and may play a role in the development of age-related chronic conditions, including metabolic, cardiovascular, and neurodegenerative diseases (Burtscher et al., 2023). Among these, FGF21 was the first mitokine identified in mammals and, along with GDF15, remains one of the most extensively studied mitokines.

**FIGURE 2:**
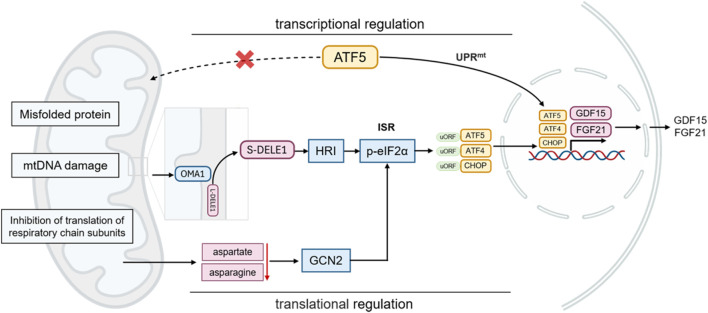
Expression and secretion of FGF21 in response to mitochondrial stress.

### 3.1 FGF21

FGF21, a mitokine, is an evolutionarily conserved endocrine metabolic regulator encoded by a gene located on human chromosome 19. It produces a 209-amino-acid secreted protein with a signal peptide. It binds to FGFR at the N-terminus and anchors to KLB at the C-terminus to form a ternary complex, initiating receptor dimerization and phosphorylation cascades that activate downstream pathways (Ding et al., 2012; Aaldijk et al., 2023; Chen et al., 2023). As a member of the endocrine FGF19 subfamily, FGF21 can be released into the bloodstream (Beenken and Mohammadi, 2009; Potthoff et al., 2012)and regulate metabolism within and between tissues *via* autocrine, paracrine, and endocrine mechanisms. While the liver is the primary source of circulating FGF21, other tissues such as the heart and adipose tissue can locally express it under stress conditions, thereby exerting protective effects through autocrine or paracrine signaling.

FGF21 is a metabolic hormone predominantly secreted by the liver, where it functions as a key energy sensor and nutrient regulator. In response to nutritional stressors such as starvation, amino acid restriction, and high-fat diet (HFD) intake, hepatic expression of FGF21 and its co-receptor KLB is upregulated, enabling the liver to adapt to diverse nutritional stimuli (Inagaki et al., 2007; Iizuka et al., 2009; Potthoff, 2017). Beyond modulating insulin activity, FGF21 also plays pivotal roles in regulating glucose and lipid metabolism, as well as facilitating adaptation to ketogenic conditions (Wang F. et al., 2021). Furthermore, acute and chronic stressors—such as exercise, oxidative stress, and fluctuations in glucose or lipid levels—have been shown to increase FGF21 levels in experimental models (Feingold et al., 2012; Gómez-Sámano et al., 2017). Studies have shown that FGF21 is also induced by mitochondrial stress and UPRmt, functioning as a mitokine to regulate systemic metabolism and promote cellular stress resistance (Ost et al., 2016).

As a dual-function hormone involved in both metabolism and cellular stress responses, FGF21 expression is regulated by a diverse array of mechanisms. Multiple transcription factors, including peroxisome proliferator-activated receptor alpha (PPARα), activating transcription factor 4 (ATF4), carbohydrate response element-binding protein (ChREBP), and CCR4-NOT transcription complex subunit 6-like protein (CNOT6L), regulate FGF21 transcription under different physiological and pathological conditions. Hepatic FGF21 can be upregulated in response to both nutrient deficiency (e.g., starvation, ketogenic diet, methionine/choline-deficient diet) and nutrient excess (e.g., high monosaccharide intake). During nutrient deprivation, fatty acids activate PPARα, which in turn induces FGF21 expression as a downstream target (Badman et al., 2007). ATF4 also promotes FGF21 expression under amino acid restriction and oxidative stress. Under conditions of carbohydrate overload, ChREBP induces FGF21 to regulate *de novo* lipogenesis in the liver and adipose tissue (Tan et al., 2023). The FGF21 promoter contains two ATF4 binding elements (AARE1 and AARE2), to which ATF4 can directly bind (Maruyama et al., 2016). Furthermore, ATF5, a transcription factor in the CREB/ATF family and closely related to ATF4 and CHOP, is implicated in stress-responsive FGF21 regulation (Yamazaki et al., 2010). In stress conditions, FGF21 contributes not only to glucose and lipid metabolism but also to the mitochondrial unfolded protein response (UPRmt), promoting cellular homeostasis through mechanisms such as reactive oxygen species (ROS) scavenging and inhibition of apoptosis (Itoh and Ohta, 2013; Planavila et al., 2015a).

### 3.2 GDF15

GDF15, also known as macrophage inhibitory cytokine-1 (MIC-1), is a member of the transforming growth factor-β (TGF-β) superfamily, GDF15 binds to the GDNF family receptor α-like (GFRAL) receptor, recruiting and activating the co-receptor RET to initiate downstream signaling pathways involved in appetite regulation and energy homeostasis (Emmerson et al., 2017; Hsu et al., 2017; Mullican et al., 2017; Yang et al., 2017). GFRAL- or GDF15-deficient mice exhibit increased food intake and weight gain, whereas exogenous GDF15 administration reduces food intake and promotes weight loss. Moreover, in obesity, membrane-bound matrix metalloproteinase 14 (MT1-MMP/MMP14) inhibits GDF15 signaling by blocking GFRAL. Animal studies demonstrate that MT1-MMP knockout restores GFRAL expression, attenuating weight gain and food intake in obese mice, suggesting this pathway as a potential therapeutic target for obesity (Chow et al., 2022).

Under normal physiological conditions, GDF15 is expressed at low levels in most organs. However, its expression is significantly upregulated in response to tissue damage or stress in organs such as the liver, kidneys, heart, and lungs. GDF15 expression is regulated by two parallel systems: the UPRmt and ISR (Costa-Mattioli and Walter, 2020; Suárez-Rivero et al., 2022a). During mitochondrial stress, UPRmt activates GDF15 transcription, while ISR modulates its expression *via* the transcription factor ATF4 (Patel et al., 2019; Kang et al., 2021; Miyake et al., 2021). Additionally, transcription factors such as ATF5 and CHOP are involved in this process. The specific cellular environment and stress type influence the activation of these transcription factors and subsequent GDF15 expression (Zhao et al., 2002; Fiorese et al., 2016). Notably, despite partial impairment of UPRmt and GDF15 regulation in ATF4, ATF5, or CHOP knockout models, other UPRmt factors and alternative mechanisms can maintain stress responses and GDF15 functionality. This complex regulatory network suggests that GDF15 expression results from the synergistic action of multiple stress pathways, with mechanisms varying by cell type and stress condition (Figure 2).

### 3.3 Mitochondria-derived peptides

Mitochondria-derived peptides (MDPs) are a novel class of microproteins encoded by mitochondrial DNA, consisting of bioactive peptides with fewer than 100 amino acids (Saghatelian and Couso, 2015; Miller et al., 2022; Kong et al., 2023). Eight MDPs have been identified, with humanin (HN) and mitochondrial open reading frame 12c-encoded peptide (MOTS-c) being the most extensively studied exercise-induced mitokines, playing pivotal roles in cellular homeostasis, cytoprotection, and metabolic regulation.

HN, a 24-amino acid polypeptide encoded by mitochondrial 16S rRNA gene, is predominantly expressed in tissues requiring high energy metabolism such as the heart, brain, liver, colon, and skeletal muscle. It exerts anti-apoptotic and antioxidant effects through three primary mechanisms: (1) activation of the PI3K/AKT signaling pathway, (2) enhancement of mitochondrial respiratory chain activity, and (3) suppression of pro-inflammatory JNK/p38 signaling pathways (Guo et al., 2003; Cai et al., 2021). MOTS-c, another critical MDP member, is regulated by mitochondrial stress responses. It improves insulin resistance and promotes metabolic homeostasis through AMPK activation *via* folate cycle inhibition. Under glucose restriction conditions, MOTS-c undergoes nuclear translocation to modulate antioxidant gene expression, thereby enhancing cellular stress resistance (Kim et al., 2018). Both MDPs demonstrate exercise-responsive expression patterns, suggesting their potential as exercise mimetics.

### 3.4 Mitokines and cardiac diseases

FGF21 and GDF15, as sensitive indicators of mitochondrial stress, are increasingly recognized for their potential to bridge interconnected pathways involving oxidative stress, chronic inflammation, and insulin resistance in cardiovascular disease research (Table 1). Elevated circulating GDF15 levels have been consistently associated with adverse outcomes in obesity-related metabolic disorders, heart failure, and atherosclerosis, underscoring their diagnostic and prognostic significance (Adela and Banerjee, 2015). While GDF15 is considered a potential prognostic biomarker, the regulatory role of its GFRAL-RET signaling pathway in cardiac tissues remains unclear‌.

**TABLE 1:** The action of FGF21 and GDF15 in cardiac diseases.

Mitokine	Source of production	Causes of stress	Effects on the heart	Stress pathway	References
FGF21	Liver	Fasting, Hunger, Protein restriction	Ketones metabolism↑ Fatty acid oxidation↑	MAPK/ERK	Badman et al. (2007), Itoh and Ohta (2013), Tan et al. (2023)
	Pancreas	Insulin resistance, Obesity	Antioxidant defense↑	Ucps/Sods	Itoh and Ohta (2013), Planavila et al. (2015a), Zhang et al. (2015), Ferrer-Curriu et al. (2021), Ma et al. (2021)
	Adipose tissue	Cold, Sympathetic excitement	Anti-apoptotic capacity↑	FGF21-p38 MAPK/AMPK	Patel et al. (2014), Joki et al. (2015), Zhang et al. (2015)
	Skeletal muscle	Mitochondrial stress Muscle atrophy	Myocardial hypertrophy↓ Myocardial fibrosis↓ Ventricular remodeling↓	MAPK, SIRT1-PGC1α, NF-κB	Itoh and Ohta (2013), Planavila et al. (2013), Xu et al. (2019), Sun et al. (2023)
	Cardiomyocytes	Myocardial infarction, Stress overload	Myocardial ischemia/reperfusion injury↓	FGFR1/KLB-PI3K-Akt1-BAD	Liu and Wu (2010), Liu et al. (2012), Liu et al. (2013)
GDF15	Heart and Cardiovascular System	Heart failure, myocardial infarction, Hypertrophic cardiomyopathy	Myocardial function↑ Myocardial hypertrophy↓	PI3K-Akt, SMAD2/3, Akt, ERK1/2	Kempf et al. (2006), Xu et al. (2006)
	Liver	Hepatitis, Hepatic Fibrosis			
	Kidney	Acute Kidney Injury, Chronic Kidney Disease	Potent anorectic action, Regulate systemic metabolic flexibility, Regulate oxidative and lipolytic functions, Regulate cardiac insulin sensitivity		Chung et al. (2017), Ost et al. (2020), Kang et al. (2021)
	Skeletal muscle	Mitochondrial stress, Intense exercise			

The antagonistic effect of FGF21 on atherosclerosis is attributed to its ability to induce adiponectin secretion in adipocytes and suppress hepatic cholesterol biosynthesis. FGF21 ameliorates atherosclerosis by inhibiting hepatic SREBP2 expression and promoting adipocyte-derived adiponectin production (Lin et al., 2015). As a regulator of mitochondrial homeostasis in cardiomyocytes under oxidative stress, FGF21’s maintenance of mitochondrial dynamics is critical for cardiomyocyte function, positioning it as a key therapeutic target for HF (Planavila et al., 2013; Planavila et al., 2015a; Yan et al., 2023). Diabetes constitutes another major cause of myocardial damage, exerting a dual assault on the heart through chronic hyperglycemia-induced coronary artery disease and direct impairment of cardiomyocyte function, thereby significantly increasing the risks of heart failure and sudden death (Marx et al., 2023). FGF21 protects against diabetic cardiomyopathy by preventing mitochondrial dysfunction *via* the AMPK/FOXO3/SIRT3 signaling axis (Jin et al., 2022). Notably, FGF21 exerts tissue-specific effects by interacting with organ-selective FGFR/KLB receptor complexes. The distribution of FGFR subtypes varies by organ: FGFR1/2 is highly expressed in adipose tissue, FGFR4 is predominantly found in the liver, and both FGFR1 and KLB are enriched in the heart (Gälman et al., 2008; Suzuki et al., 2008; Fon Tacer et al., 2010; Yang et al., 2012; Planavila et al., 2013; Li, 2019). This spatial receptor distribution underpins the FGF21-mediated metabolic network, positioning FGF21 as a pivotal signaling hub that coordinates cross-tissue reprogramming of glucose and lipid metabolism (Fon Tacer et al., 2010; Moure et al., 2021). Therefore, taking FGF21 as a focal point, this provides new support for exploring moderate exercise and dietary strategies that promote healthy aging and cardiac rehabilitation. In the next section, we will use FGF21 as an example to investigate the role of UPRmt-regulated mitokines in cardiac diseases.

## 4 The action of FGF21 in cardiac diseases

Under cardiac stress conditions, FGF21 is expressed and secreted by cardiomyocytes, where it exerts local autocrine and paracrine effects. FGF21 produced by cardiomyocytes protects against hypertrophic damage (Planavila et al., 2013) and functions as an antioxidant within the heart, preventing the accumulation of ROS through autocrine signaling (Itoh and Ohta, 2013). In addition, fibroblast growth factors (FGFs) released *via* paracrine and endocrine mechanisms have been shown to exert anti-hypertrophic, antioxidative, and anti-apoptotic effects under both physiological and pathological conditions (Itoh and Ohta, 2013; Liu et al., 2013; Planavila et al., 2015a). These findings suggest that, beyond its metabolic regulatory roles, FGF21 also functions as a stress-responsive factor critical for maintaining cardiomyocyte homeostasis.

Various physiological conditions—such as fasting (Fazeli et al., 2015), high sugar intake (Lundsgaard et al., 2017), and dietary protein restriction (Laeger et al., 2014)—can alter circulating FGF21 levels in humans. However, some studies indicate that a ketogenic diet does not significantly affect plasma FGF21 concentrations in humans (Christodoulides et al., 2009). In diet-induced obesity models, increased FGF21 expression appears to be associated with multiple factors, including organelle stress (such as endoplasmic reticulum and mitochondrial stress) (Kim and Lee, 2014) and the phenomenon of FGF21 resistance (Fisher et al., 2010). While FGF21 is detectable in muscle biopsies under normal conditions (albeit at lower levels than in the liver) (Fisher and Maratos-Flier, 2016), stressed skeletal muscle tissue can significantly upregulate and secrete FGF21 (Dogan et al., 2014; Salminen et al., 2017; Forsström et al., 2019). Recent findings further demonstrate that the heart functions not only as a source of FGF21 but also as a target tissue (Planavila et al., 2013; Jovaisaite and Auwerx, 2015).

Preclinical research indicates that FGF21 plays a bidirectional regulatory role in pathological states such as myocardial infarction, pressure-overload-induced cardiac hypertrophy, and heart failure. The heart serves both as a site of FGF21 synthesis—where cardiomyocytes secrete it *via* the SIRT1-PPARα signaling pathway—and as a major target tissue, with high local expression of the FGFR1/KLB receptor complex (Planavila et al., 2013; Planavila et al., 2015b; Tucker et al., 2023). In acute myocardial ischemia models, FGF21 activates the FGFR1/KLB-ERK signaling pathway in cardiomyocytes, leading to phosphorylation of CREB and upregulation of PGC1α, forming a protective regulatory cascade. PGC1α in turn suppresses NF-κB-mediated inflammatory responses and enhances fatty acid oxidation. It also induces the expression of mitochondrial antioxidant proteins such as UCP3 and SOD2, collectively reducing ROS accumulation and improving mitochondrial function (Planavila et al., 2013; Planavila et al., 2015a; Zhang et al., 2015). Additionally, activation of the FGF21–p38 MAPK/AMPK pathway can inhibit apoptotic signaling, thereby attenuating ischemia-reperfusion injury and myocardial fibrosis (Patel et al., 2014; Joki et al., 2015; Zhang et al., 2015).

In chronic pathological models, endogenous FGF21 provides compensatory and protective effects through regulation of the heart-liver metabolic axis. Under pressure overload, cardiac fibroblasts secrete FGF21 *via* a DPP-4 inhibitor-sensitive pathway, acting on cardiomyocytes in a paracrine manner to enhance stress resilience and confer cardioprotection. Moreover, hepatic congestion associated with heart failure with preserved ejection fraction (HFpEF) induces hepatic FGF21 expression, which in turn regulates cardiac metabolism as part of a compensatory protective mechanism—constituting a protective feedback loop between organs (Furukawa et al., 2021; Tucker et al., 2023). Gene knockout studies have confirmed that cardiac-specific deletion of FGF21 disrupts the myocardial antioxidant defense system, as evidenced by reduced UCP3/SOD2 expression, increased ROS accumulation, and aggravated cardiomyocyte apoptosis and pathological remodeling (Ferrer-Curriu et al., 2021; Ma et al., 2021). Pharmacological studies have shown that administration of exogenous FGF21 at supraphysiological concentrations markedly improves conditions such as obesity, insulin resistance, and nonalcoholic fatty liver disease. However, the metabolic effects of FGF21 exhibit species-specific differences: while rodents experience significant weight loss, humans show only modest improvements (Xu et al., 2009; Fisher et al., 2011; BonDurant et al., 2017; Geng et al., 2020).

Importantly, the cardioprotective effects of FGF21 appear to be dose-dependent. At physiological concentrations, FGF21 primarily maintains mitochondrial homeostasis through the UPRmt mechanism, whereas at elevated concentrations (such as those released from endothelial cells during ischemia), it reduces infarct size by suppressing inflammatory and oxidative stress cascades (Liu et al., 2013; Patel et al., 2014). These findings underscore FGF21’s role as a central regulator of cardiac metabolic and redox homeostasis, offering new insights and directions for understanding its function in inter-organ signal communication.

However, controversies remain regarding FGF21’s physiological and pathological roles. Elevated circulating FGF21 levels have been observed not only in patients with heart failure but also in those with coronary heart disease (Lin et al., 2010). In Opa1^−/−^ mice with mitochondrial dysfunction, serum FGF21 levels are significantly elevated. Interestingly, while skeletal muscle in these mice shows upregulated expression of KLB and FGFR, no notable changes are observed in other tissues (Tezze et al., 2017). Moreover, in mice with mitochondrial respiratory chain deficiencies, both skeletal muscle-derived FGF21 (SM-FGF21) mRNA and serum FGF21 levels are increased (Tyynismaa et al., 2010). Correspondingly, elevated serum FGF21 has been detected in humans with mitochondrial respiratory chain defects in muscle, suggesting its potential utility as a biomarker for diagnosing mitochondrial myopathies (Suomalainen et al., 2011). Mechanistic studies indicate that ATF4 may drive FGF21 overexpression and mitochondrial dysfunction in skeletal muscle, potentially mediated by excessive ROS production (Kim et al., 2013). Although obese individuals often exhibit elevated serum FGF21 levels, these increases are not associated with metabolic improvements. Some animal studies propose that reduced expression of FGFR1 and KLB in adipose tissue may impair FGF21 signaling, a phenomenon referred to as “FGF21 resistance” (Markan, 2018).

It is important to note that the current literature does not clearly distinguish between the physiological functions of endogenous FGF21 and the pharmacological effects of exogenous FGF21. Many studies exploring FGF21’s pharmacological effects employ Fgf21 transgenic mouse models or administer FGF21 at supraphysiological doses. Such experimental approaches may partly explain the observed discrepancies between endogenous and exogenous FGF21 effects, while also underscoring the critical knowledge gaps that remain in elucidating the functional networks of FGF21 and other mitochondrial regulatory factors.

## 5 Inter-organ communication of FGF21 and cardiac diseases

In a mouse model of myocardial ischemia, the liver responds by upregulating the expression and release of cardioprotective proteins such as FGF21, contributing to cardiac protection during myocardial infarction (Liu and Wu, 2010). Recent studies using microarray gene expression and proteomic profiling have demonstrated elevated FGF21 protein levels in both hepatic and adipose tissues following myocardial infarction in mice. Similarly, in ischemia/reperfusion (I/R) injury models, hepatocytes increase FGF21 expression and release it into circulation, where it interacts with the FGFR1/KLB receptor complex on cardiomyocytes to initiate the cardioprotective FGFR1/KLB–PI3K–Akt1–BAD signaling cascade. Furthermore, FGF21 accumulation is observed in diseased myocardial tissue, and serum FGF21 levels show a strong positive correlation with cardiac FGFR3 expression. These findings support the notion that hepatocyte-derived FGF21 exerts endocrine-mediated protection on ischemic cardiomyocytes (Liu and Wu, 2010; Liu et al., 2012; Liu et al., 2013). Inhibition of mineralocorticoid receptor (MR) expression or treatment of hepatocytes with the MR antagonist spironolactone has been shown to enhance FGF21-mediated cardiac repair and reverse pathological remodeling following myocardial infarction (Greenberg et al., 2006; Sun et al., 2023). These findings suggest that liver-derived endocrine FGF21 plays a crucial role in alleviating myocardial ischemic injury and may provide new avenues for targeting UPRmt to promote cardiac recovery post-infarction.

In response to acute or chronic exercise, multiple tissues including the liver, brain, heart, pancreas, intestine, and adipose tissue release hundreds of exercise-induced factors. Key cytokines secreted by muscle fibers include FGF21, irisin, interleukin-6 (IL-6), interleukin-15, apelin, actin, and myonectin (Thyfault and Bergouignan, 2020). Aerobic exercise, such as structured training in mice, induces FGF21 expression in skeletal muscle, and the endocrine FGF21 entering circulation has been shown to exert cardioprotective effects (Yan et al., 2017). Increasing evidence suggests that mitokines induced by moderate exercise may mitigate metabolic risk factors associated with heart failure. Experimental and multi-omics studies on physical exercise indicate that mitokines from skeletal muscle and other tissues regulate cardiac function *via* endocrine mechanisms (Jin et al., 2024). Under basal physiological conditions, skeletal muscle is not considered the primary source of FGF21 (Fon Tacer et al., 2010). However, although liver-derived FGF21 is dominant in humans, in mice skeletal muscle may significantly contribute to circulating FGF21 levels during exercise (Tezze et al., 2019), suggesting a potential role of muscle in FGF21 secretion.

In addition to exercise, factors such as fasting, insulin, and mitochondrial stress can also induce FGF21 expression in skeletal muscle, highlighting mitochondrial stress as a key stimulus for increased FGF21 production in humans (Izumiya et al., 2008; Kim et al., 2013; Keipert et al., 2014; Pereira et al., 2017; Tezze et al., 2017). Upregulation of FGF21 expression has also been observed in mitochondrial dysfunction models involving suppression of mitochondrial fusion factor optic atrophy one and mitochondrial DNA stress in mitochondrial myopathy (Pereira et al., 2017; Tezze et al., 2017; Forsström et al., 2019). Moreover, skeletal muscle-derived FGF21 has been shown to modulate cardiac remodeling in mouse models of myocardial infarction (Joki et al., 2015). Exercise training has been demonstrated to reduce cardiac fibrosis induced by a high-fat diet (Yan et al., 2017). In mouse skeletal muscle, the upregulation and secretion of FGF21 depend on activation of the phosphatidylinositol 3-kinase (PI3K)/Akt1 signaling pathway (Izumiya et al., 2008; Keipert et al., 2014). FGF21-mediated adaptive responses to metabolic stress in skeletal muscle are therefore regarded as key regulatory mechanisms in disease progression and metabolic control (Baskin et al., 2015). Additionally, FGF21-mediated nucleocytoplasmic signaling reciprocally influences mitochondrial function, reinforcing the notion that mitokines collectively contribute to muscle mass maintenance, attenuation of hypertriglyceridemia, and improved insulin sensitivity.

**FIGURE 3:**
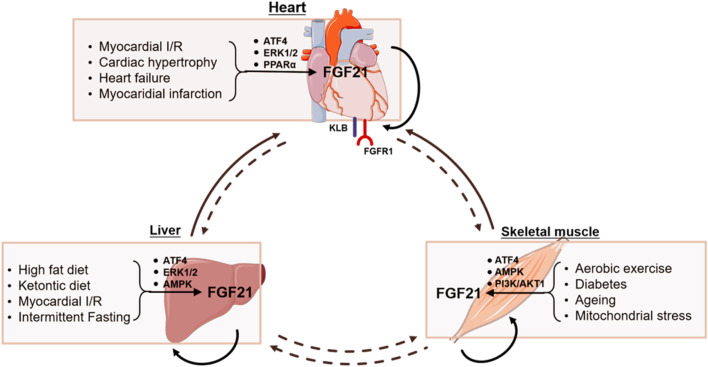
FGF21 generation and its role in interorgan crosstalk.

To enhance the effectiveness of cardiac rehabilitation, researchers have begun to explore how mechanical stress in skeletal muscle and localized changes in temperature, oxygen consumption, and metabolism regulate mitokines such as FGF21. This research aims to uncover the mechanisms underlying cross-talk between the cardiovascular, respiratory, immune, and nervous systems, and the broader physiological effects of these interactions (Burtscher et al., 2021; Jin et al., 2024). Such insights will further advance the field of mitochondrial pathophysiology (Figure 3). A deeper understanding of how UPRmt-induced factors like FGF21 are modulated under various physiological and pathological conditions—and how they affect systemic health—may offer new therapeutic strategies to harness their beneficial effects and promote healthy aging.

## 6 Application potential of targeting UPRmt/FGF21

The UPRmt and ISR have emerged as promising therapeutic targets for mitochondrial diseases, owing to their central roles in maintaining cellular metabolic homeostasis and the elucidation of their molecular mechanisms. ging evidence demonstrates that diverse compounds can modulate the UPRmt or related signaling pathways. For instance, tetrahydrocurcumin (THC), the main metabolite of curcumin, activates the UPRmt through the PGC-1α/ATF5 axis, thereby reducing reactive oxygen species (ROS) production, improving mitochondrial dysfunction, and preventing pathological cardiac hypertrophy (Zhang et al., 2020). Pterostilbene, a structural analog of the sirtuin activator resveratrol, has also been shown to activate the UPRmt *via* the SIRT/FOXO3a/PGC1α/NRF1 signaling pathway and alleviate pathological changes associated with mitochondrial dysfunction (Germain, 2016; Suárez-Rivero et al., 2022b). Recent *in vivo* research demonstrated that administration of doxycycline, a UPRmt activator that acts through ATF5, offers cardioprotective effects in a murine model of ischemia/reperfusion (I/R) injury (Wang et al., 2019). Other studies indicate that choline improves mitochondrial function *via* the SIRT3/AMPK/UPRmt axis, thereby inhibiting myocardial hypertrophy in mice (Xu et al., 2019). In neurodegenerative models, nicotinamide riboside (NR) has been shown to activate the UPRmt, helping to maintain mitochondrial protein homeostasis and mitigate neurodegenerative phenotypes in mice with amyotrophic lateral sclerosis (ALS) (Zhou et al., 2020). These findings collectively suggest that targeting the UPRmt holds therapeutic potential (Table 2).

**TABLE 2:** Representative drugs with potential UPRmt-activating effects.

Treatment	Targets	Pathological condition	Health benefits/effects	References
Tetrahydrocurcumin	Activate PGC1α/ATF5 axis	Pathological Cardiac Hypertrophy	Resist pathological cardiac hypertrophy and improve mitochondrial function	Zhang et al. (2020)
Pterostilbene	Increase the NAD^+^/NADH ratio and Sirt3 activity	Mitochondrial diseases	Improve pathological alterations in mutant fibroblasts and induced neurons	Suárez-Rivero et al. (2022b)
Choline	Activate SIRT3-AMPK pathway	Ventricular hypertrophy	Preserve the ultrastructure and function of mitochondria in the context of cardiac hypertrophy; attenuate cardiac dysfunction	Xu et al. (2019)
Nicotinamide ribose	Activate NAD^+^/Sirtuins pathway	Amyotrophic lateral sclerosis	Modulate mitochondrial proteostasis and improve the adult neurogenesis in the brain of SOD1^G93A^mice	Zhou et al. (2020)

However, the clinical translation of these compounds is still hindered by significant limitations, including low bioavailability and off-target effects. THC and pterostilbene suffer from poor oral bioavailability and metabolic instability; choline, as a nutritional supplement, lacks specificity in its effects, making it difficult to attribute to the specific activation of UPRmt; NR undergoes complex metabolism *in vivo*, potentially affecting multiple NAD^+^-dependent pathways; and long-term use of doxycycline as an antibiotic may lead to resistance and microbiome dysbiosis. Although preclinical studies have demonstrated their potential therapeutic effects, there remains a lack of reliable drugs capable of precisely modulating the UPRmt pathway. Therefore, advancing the development of therapeutic strategies targeting UPRmt requires further exploration of compound optimization, delivery strategies, and rigorous safety evaluations.

Recent laboratory and clinical studies have increasingly revealed the cardioprotective effects of FGF21. Therapeutic strategies for cardiovascular diseases based on FGF21 primarily revolve around its pleiotropic metabolic regulatory functions. Numerous long-acting FGF21 analogs and monoclonal antibodies that agonize the FGFR1-KLB receptor complex have also been developed. Due to the effects of FGF21 analogs on parameters such as blood pressure and heart rate, current research in cardiovascular diseases remains largely confined to the preclinical stage. Multiple FGF21 analogs (e.g., Pegbelfermin, Efruxifermin) and receptor agonists (e.g., MK-3655) indirectly protect cardiac function by improving systemic insulin sensitivity, reducing inflammation, and alleviating lipotoxicity. Particularly in diabetic cardiomyopathy, these drugs can ameliorate myocardial metabolic disorders, inhibit fibrosis progression, while their triglyceride-lowering effects (e.g., PF-05231023) and lipid profile improvements help mitigate atherosclerotic burden (Chen et al., 2025).

To date, six randomized clinical trials have evaluated the therapeutic potential of four human FGF21 analogs or mimetics in T2DM or obesity (Zhang et al., 2024). FGF21 demonstrates direct cardioprotective effects and potential therapeutic prospects for improving myocardial energetics and function in obesity and T2DM through its pleiotropic actions (metabolic improvement, inflammation reduction, and fibrosis suppression), with related drugs currently in clinical development. Future development of targeted delivery systems for FGF21 analogs, as well as the advancement of tissue-selective FGF21 receptor agonists and FGF21 sensitizers, may enhance the efficacy and safety of FGF21-based therapies.

## 7 Conclusions and prospects

The mitochondrial unfolded protein response (UPRmt) plays a vital role in maintaining mitochondrial homeostasis and metabolic balance in cardiomyocytes. Evidence from animal studies suggests that certain pharmacological agents can alleviate cardiovascular diseases by activating stress response pathways, highlighting the potential of UPRmt and its associated mitokines as novel therapeutic targets. Based on current research, several key issues need to be addressed in future studies: ① The threshold between the protective and deleterious effects of UPRmt remains unclear. Although some molecular markers have been identified, further investigation is needed to determine which markers are most suitable for evaluating UPRmt activity. ② With the aid of advanced research technologies, the regulatory interactions between UPRmt and mitokines—both at the interorgan and intercellular levels—require more in-depth exploration. ③ The relationship between the integrated stress response (ISR) and UPRmt is still not fully understood. Further analysis of the intersecting signaling pathways that activate or influence UPRmt is needed. Continued research in these areas is expected to provide robust evidence to support the development of UPRmt- and mitokine-targeted therapies, deepen our understanding of their roles in cardiac function regulation, and ultimately contribute to improved treatment and rehabilitation strategies for heart disease.
